# Tributyltin-mediated hepatic, renal and testicular tissue damage in male Syrian hamster (*Mesocricetus auratus*): a study on impact of oxidative stress

**DOI:** 10.1186/s40064-016-3186-1

**Published:** 2016-09-09

**Authors:** V. Kanimozhi, K. Palanivel, M. A. Akbarsha, B. Kadalmani

**Affiliations:** 1 Department of Animal Science, Bharathidasan University, Tiruchirappalli, 620 024 India; 2 Department of Food Science and Nutrition, College of Food and Agriculture, King Saud University, Riyadh, Kingdom of Saudi Arabia

**Keywords:** Tributyltin, Organotin, Antioxidant enzymes, Serum markers

## Abstract

Organotin compounds are a versatile group of organometallic chemicals that are used in a variety of industrial and agricultural applications. Tributyltin (TBT), a common organotin, brings about severe spermatotoxic and organotoxic effects. However, information about the adverse effects of TBT on liver, kidney and testis is scanty. Hence, the present study was undertaken to elucidate the TBT-mediated oxidative stress-induced impairments in these organs. Administration of TBT through oral route at increasing doses of 50, 100 and 150 ppm for 65 days to male Syrian hamsters resulted in drastically decreased activities of antioxidant enzymes superoxide dismutase, catalase and glutathione peroxidase and decreased mean levels of non-enzymatic antioxidants (reduced glutathione, vitamin C, and vitamin E) followed by a dramatic increase in the levels of lipid peroxidation in the liver, kidney and testis as compared to the control animals. Significantly high levels of serum urea, creatinine, uric acid and bilirubin were observed in TBT-treated hamsters. Also, TBT treatment induced drastic histopathological changes in the liver, kidney and testis combined with remarkable changes in serum levels of tissue injury marker enzymes Aspartate transaminases, Alkaline phosphatase and Alanine transaminase. These data affirm that exposure to TBT can lead to oxidative stress-induced damage to liver, kidney and testis.

## Background

The organotin tributyltin (TBT) is a widely used component of paints, which has been reported to mount up in fish and marine wildlife due to extensive harbor activities, fishing, etc. Several studies have reported that high levels of TBT persist in coastal waters and seafoods (Jiang et al. 2001). TBT has been evidenced to bioaccumulate as conjugates in organisms with biomagnification in the food web and thus affecting human health and environment. Among the various possible sources of contamination, metal-based antifouling agents evidently contribute greatly (Loganathan et al. 2001). TBT is commonly used as a biocide in antifouling paints and as wood preservatives. It is also used as an antifungal agent in textiles, industrial water systems (such as cooling towers and refrigeration water systems), wood pulp, paper mill systems, and breweries (Kimbrough 1976). TBT is a toxic contaminant of marine bionetwork causing irreversible damage to the marine life (Zuo et al. 2012; Revathi et al. 2013). Several studies report that TBT inflicts immunotoxicity, reproductive toxicity, embryotoxicity, genotoxicity (Antizar-Ladislao 2008) and endocrine disrupting effects (Lagadic et al. 2007). Accretion of industrial effluents and agricultural run-offs in water bodies has turned out to be the most important disquiet in freshwater ecosystems (FAO 1986). TBT exposure, been reported to have negative impact on survival, growth, reproduction, and development of marine species (Gibbs and Bryan 1986; Maguire 1987). However, the potential effect of TBT toxicity and impairment on male fertility of marine wildlife is not yet clearly known (Lewis and Ford 2012). The current and upcoming restrictions will not immediately remove TBT and its degradation products from the marine environment, since these compounds are persistent in the sediments. Hence, article was aimed at finding the impact of TBT in male Syrian hamster as the model organism adopting biochemical and histological analysis of major target organs with a view that the outcome can potentially reflect in the biological processes such as growth and reproduction. This can also throw light on early effects of the toxicant in/on the cells of organs such as liver, kidney, testis, etc. (Hinton et al. 1992; Mohan Raj 2007). Biochemical parameters are considered to be the best early indicators of oxidative stress condition caused by xenobiotics (Emmanouil et al. 2008). Antioxidant reactions of living organisms are the central defense mechanisms recruited to circumvent the effects of toxicants. Several reports suggest that these responses can be used as biomarkers that reflect pollution levels of such toxic substances (Huang et al. 2005a, b). Enzymatic anti-oxidants such as SOD, CAT and GPx, and non-enzymatic antioxidants such as GSH, vitamin C and vitamin E constitute the antioxidant defense system against reactive oxygen species (ROS) in the cells whereas AST, ALP, and ALT are the serum indicators of tissue injury.

This study was designed to address the issue of TBT toxicity and its direct correlation with antioxidant responses and bioaccumulation under experimental conditions.

## Methods

### Chemicals

TBT chloride was purchased from Sigma Aldrich Chemical Co. (purity > 97 %; St. Louis, MO, USA). TBT was suspended in deionized water to obtain final concentrations of 50, 100 and 150 ppm. All other chemicals were of analytical grade and obtained from Medox Biotech, Chennai, India.

### Animal experimentation

Six to seven week old male Syrian hamsters were used in the study, and the experimental protocol was approved by the Institutional Animal Ethics Committee (BDU/IAEC/2012/76/28.03.2012). The animals were housed under 12 h light/12 h dark cycle and controlled conditions (ambient temperature 21 ± 2 °C; humidity 51 ± 7 %) and fed standard pellet feed (Purchased from Sai Enterprises, Chennai) and water ad libitum. Food and water consumption of the animals were measured daily. The body weight was recorded on day 0 and at the end of the experimental period. The hamsters were randomly divided into 4 groups, each containing five animals. Three of the four groups were used as treatment groups and one as the control group. TBT was administered through oral route at a dosage of 50, 100 or 150 ppm/kg/day for 65 days. The control animals received deionized water. At the end of the experiment the animals were immobilized by mild chloroform anesthesia and blood was drawn by cardiac puncture and serum separated by centrifugation (for 10 min at 3000 rpm). Further the animals were euthanized with excess of sodium pentobarbital followed by decapitation as a secondary physical method of euthanasia. The collected serum was used for biochemical studies. The right testis, right kidney and right lobe of the liver were dissected and fixed in Bouin’s fluid for histological analysis and the remaining tissues were frozen at −20 °C until further analysis.

### Preparation of liver, kidney and testis tissue homogenate

Liver, kidney and testis tissue from each experimental animal was homogenized (100 mg/ml buffer) with 50-mM phosphate buffer (pH 7.0) and centrifuged at 12,000*g* for 15 min at 4 °C. The supernatant thus obtained was used for biochemical assays.

### Antioxidant enzymes assay

#### SOD activity

The activity of SOD was assayed according to the method of Murkland and Murkland (1974). Enzyme activity was measured in an assay mixture containing 2 ml of Tris–HCl (pH 8.2), 2 ml distilled water, 0.5 ml tissue homogenate, and 0.5 ml 2 mM pyrogallol. The resulting color was read immediately at 470 nm at 1 min intervals for 3 min in a spectrophotometer against a blank containing all components except the sample preparation and pyrogallol. The enzyme activity was expressed as units/mg protein.

#### CAT activity

CAT activity in the tissue homogenate was assayed by the method of Sinha (1972). In this assay, dichromatic acetic acid was reduced to chromic acetate when heated in the presence of hydrogen peroxide (H_2_O_2_), with the formation of perchloric acid as an unstable intermediate. In the test reaction, the green color developed was read at 590 nm against blank in a spectrophotometer. The activity of CAT was expressed as micromoles of H_2_O_2_ consumed/min/mg protein.

#### GPx activity

The activity of GPx in the tissue homogenate was determined as described by Rotruck et al. (1973). The underlying principle is to measure the rate of glutathione oxidation, as catalyzed by the GPx present in the supernatant; the color that develops is read against a reagent blank at 412 nm.. The activity of GPx was expressed as micrograms of GSH consumed/min/mg protein.

### Levels of non-enzymatic antioxidants

The levels of GSH were assayed according to the method of Moron et al. (1979). To the tissue homogenate 0.5 ml of 10 % trichloroacetic acid was added, and the mixture was centrifuged. To the protein-free supernatant, 4 ml of 0.3 M Na_2_HPO_4_ (pH 8.0) and 0.5 ml of 0.04 % (w/v) 5,5-dithiobis-2-nitrobenzoic acid were added. The absorbance of the resulting yellow colored mixture was read in a spectrophotometer at 412 nm. The results were expressed as micrograms per milligram tissue. Vitamin C in the tissue homogenates was measured by the method of Omaye et al. (1979). Vitamin C is oxidized by copper to form dehydroascorbic acid which reacts with 2,4-dinitrophenyl hydrazine to form bis-2,4-dinitrophenyl hydrazine; this undergoes further rearrangement to form a product with an absorption maximum at 520 nm. The results were expressed as micrograms per milligram tissue. Vitamin E in the tissue extracts was estimated by the method of Desai (1984). In this method, ferric ions are reduced to ferrous ions in the presence of tocopherol, resulting in the formation of a pink color, which was read spectrophotometrically at 536 nm. The results were expressed as micrograms per milligram tissue.

### Lipid peroxidation in tissue samples

The mean concentration of malondialdehyde (MDA), a measure of lipid peroxidation, was assayed in the form of thiobarbituric acid-reacting substances (TBARS). Briefly, to 0.2 ml of 8.1 % sodium dodecyl sulphate, 1.5 ml of 20 % acetic acid (pH 3.5) and 1.5 ml of 0.81 % aqueous solution of thiobarbituric acid were added in succession. To this reaction mixture, 0.2 ml of the tissue homogenate was added. The mixture was then heated in boiling water for 60 min. After cooling to room temperature, 5 ml of butanol:pyridine (15:1 v/v) solution was added. The mixture was then centrifuged at 5000 rpm for 15 min. The upper organic layer was separated, and the intensity of the resulting pink color was read at 532 nm in a spectrophotometer. Tetramethoxypropane was used as the external standard. The level of lipid peroxides was expressed as nmoles of MDA formed/mg protein.

### Determination of activities of serum marker enzymes of tissue injury

Activities of AST and ALT in the serum were determined according to King (1965a, b) and that of ALP according to King (1965a, b) and expressed as IU/L.

### Determination of serum biochemical parameters

Serum levels of albumin, bilirubin, uric acid, creatinine and urea were determined using Ecoline Kits (Merck India Pvt. Ltd.), in an autoanalyzer (Selectra Junior, Merck India Pvt. Ltd.).

### Histopathology

Testis, kidney and liver tissues, fixed for 14–18 h in Bouin’s fixative, were processed in a graded series of ethanol solutions, and embedded in paraffin. Sections were cut in a microtome at 5 μm thickness, stained with hematoxylin and eosin and mounted in DPX mountant. The sections were viewed in a light microscope (Olympus BX51, Tokyo, Japan) and photomicrographs were obtained in the camera attachment to the microscope (Olympus C-5050, Olympus Optical Co. Ltd., Japan).

### Statistical analysis

The values presented in the bar diagrams represent the means (M) ± standard deviations (SD) of data from five animals each. The significance of difference between control and experimental groups was assessed by one-way analysis of variance (ANOVA) using SPSS software package for Windows (Version 16.0; SPSS Inc., Chicago, IL, USA). Post-hoc test was performed for inter-group comparisons (between two groups) using the least significant difference (LSD) test. Values were considered statistically significant when p < 0.05 and p < 0.001.

### Results

#### Effect of TBT on enzymatic antioxidants

**Fig. 1:**
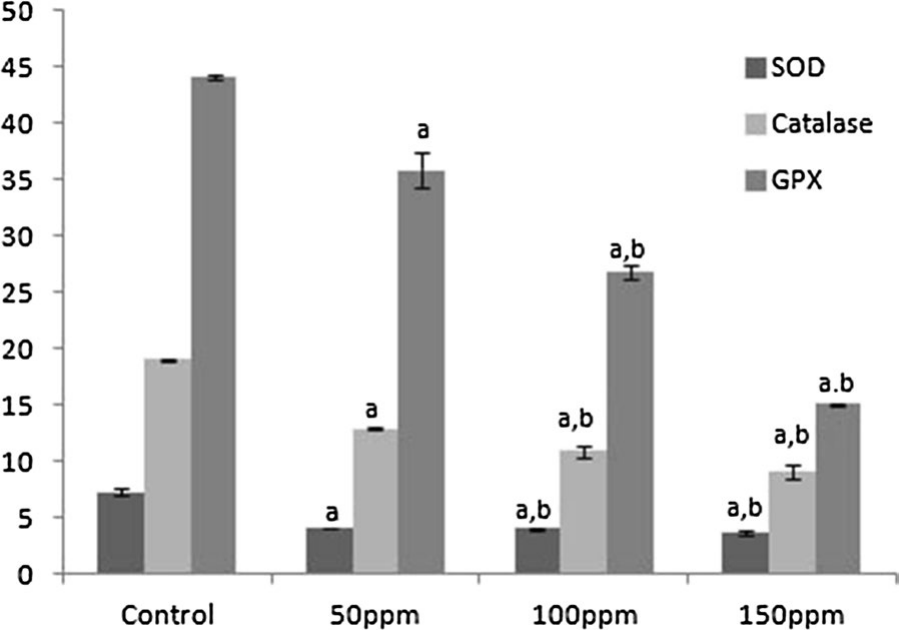
Activity levels of enzymatic antioxidants in liver tissue of Syrian hamster. Each value represents the mean ± SD of data from five animals. The enzyme activities are expressed as: SOD—units per milligram protein; CAT—micromoles of H_2_O_2_ consumed/min/mg protein; GPx—micrograms of reduced glutathione consumed/min/mg protein. ^a^Difference with the control is statistically significant (p < 0.001); ^b^Difference between treatment groups is statistically significant (p < 0.05)

The activities of SOD, CAT and GPX in the liver, kidney and testis of hamsters in all three TBT-treated groups decreased to significant levels (p < 0.001) compared to controls in a dose-dependent manner. The values between the different dose points were also significantly di fferent (p < 0.05) (Figs. 1, 4, 7).

#### Effect of TBT on non-enzymatic antioxidants

**Fig. 2:**
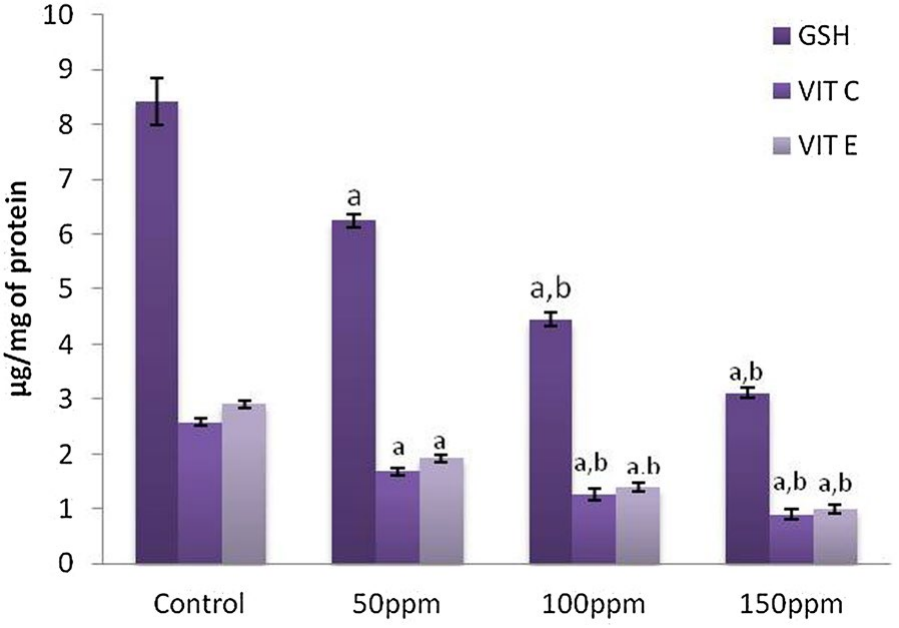
Levels of non-enzymatic antioxidants in liver tissue of Syrian hamster. Each value represents the mean ± SD of data (μg/mg of tissue) from five animals. ^a^Difference with the control is statistically significant (p < 0.001); ^b^Difference between treatment groups is statistically significant (p < 0.05)

The levels of GSH, vitamin C and vitamin E in the liver, kidney and testis of hamsters in 50 ppm (Group II),100 ppm (Group III) and 150 ppm (Group III) TBT-treated groups decreased to significant levels (p < 0.001) compared to control group of animals (Group I) in a dose-dependent manner. The values between different dose groups also were significantly different (p < 0.05) (Figs. 2, 5, 8).

#### Effect of TBT on lipid peroxidation

**Fig. 3:**
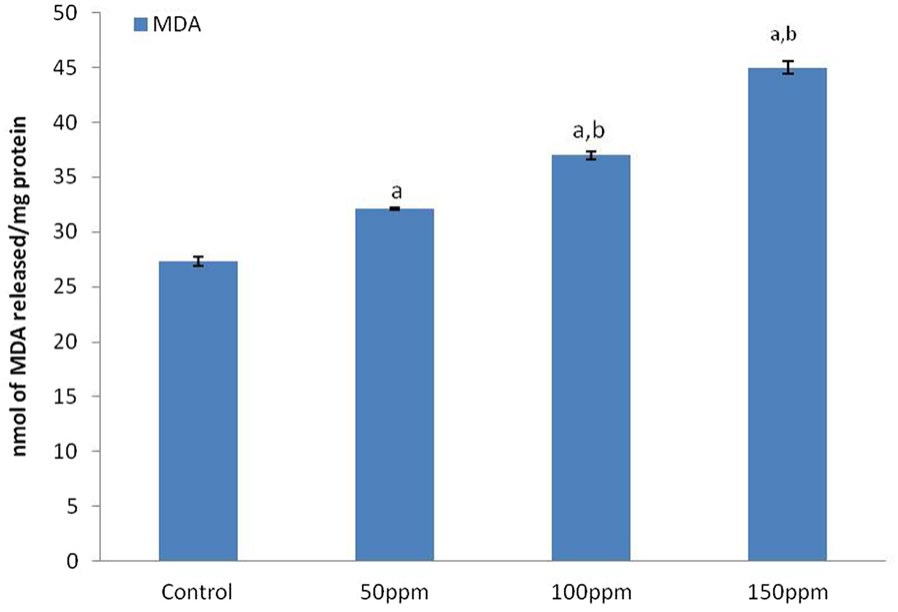
Level of malondialdehyde (MDA) in liver tissue of Syrian hamster. Each value represents the mean ± SD of data (nmoles of MDA formed/mg protein) from five animals. ^a^Difference with the control is statistically significant (p < 0.001); ^b^Difference between treatment groups is statistically significant (p < 0.05)

Significantly (p < 0.001) higher MDA levels were recorded in samples of liver, kidney and testis from 50, 100 and 150 ppm TBT-treated groups relative to the values in control animals. The values between treatment groups also were significant at p < 0.05 (Figs. 3, 6, 9).

### Activities of serum marker enzymes of tissue injury

**Fig. 4:**
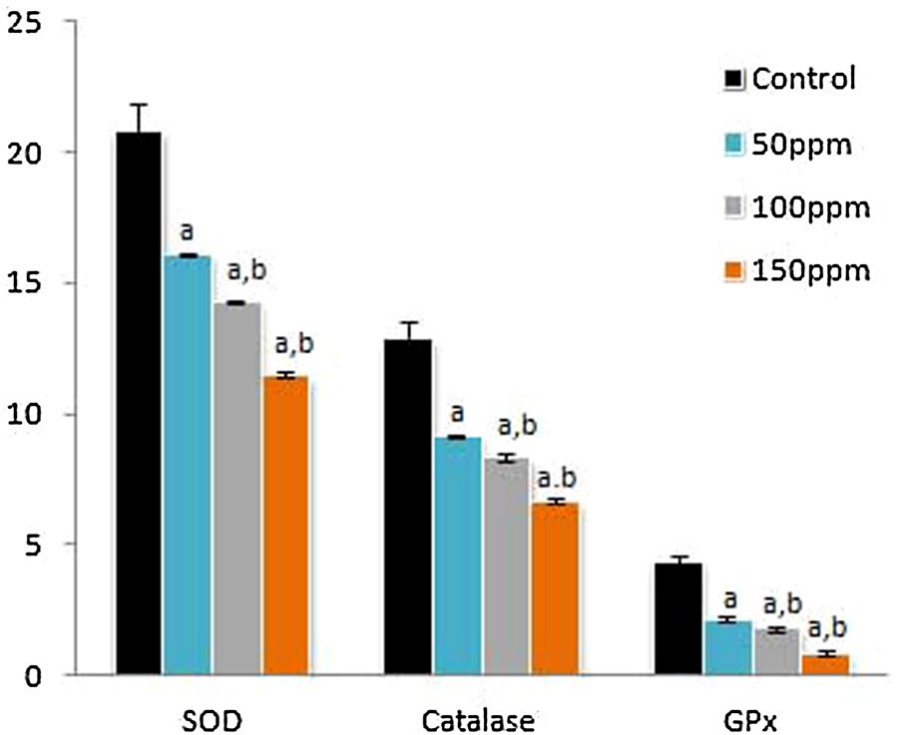
Activity levels of enzymatic antioxidants in kidney tissue of Syrian hamster. Each value represents the mean ± SD of data from five animals. The enzyme activities are expressed as: SOD—units per milligram protein; CAT—micromoles of H_2_O_2_ consumed/min/mg protein; GPx—micrograms of reduced glutathione consumed/min/mg protein. ^a^Difference with the control is statistically significant (p < 0.001); ^b^Difference between treatment groups is statistically significant (p < 0.05)

**Fig. 5:**
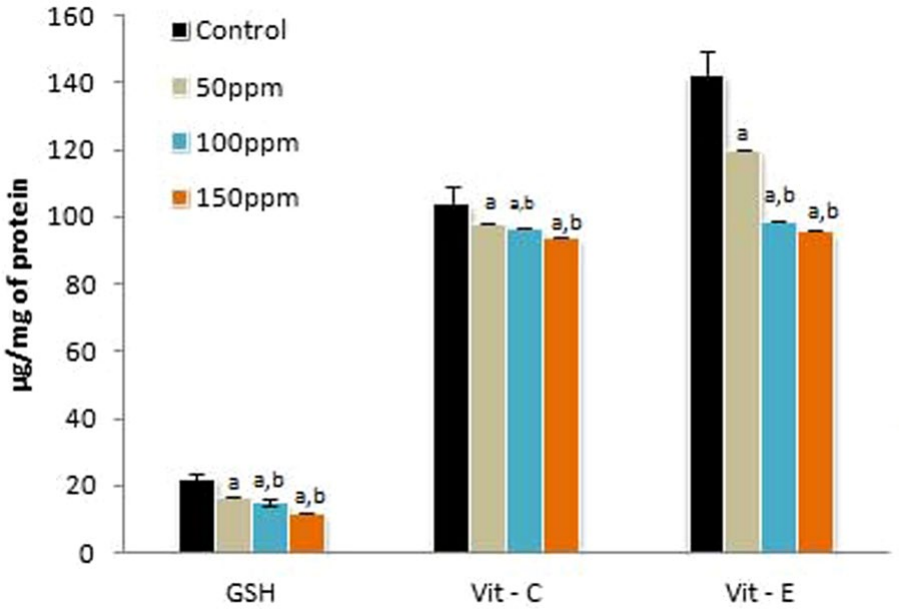
Levels of non-enzymatic antioxidants in kidney tissue of Syrian hamster. Each value represents the mean ± SD of data (μg/mg of tissue) from five animals. ^a^Difference with the control is statistically significant (p < 0.001); ^b^Difference between treatment groups is statistically significant (p < 0.05)

**Fig. 6:**
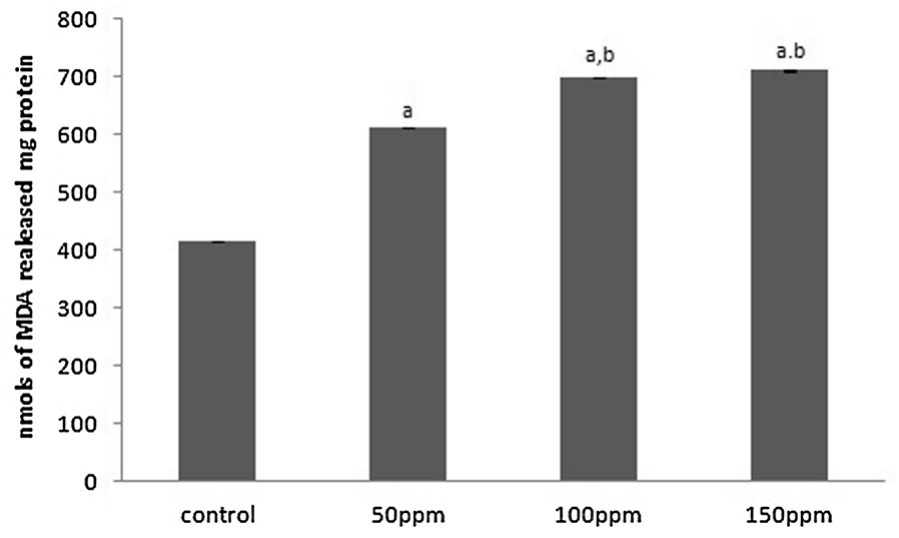
Level of malondialdehyde (MDA) in kidney tissue of Syrian hamster. Each value represents the mean ± SD of data (nmoles of MDA formed/mg protein) from five animals. ^a^Difference with the control is statistically significant (p < 0.001); ^b^Difference between treatment groups is statistically significant (p < 0.05)

**Fig. 7:**
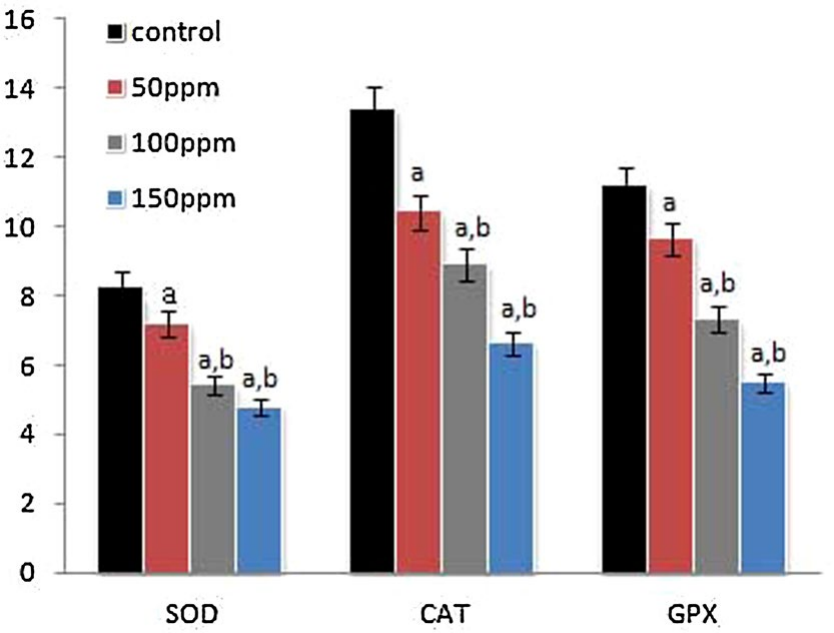
Activity levels of enzymatic antioxidants in testis tissue of Syrian hamster. Each value represents the mean ± SD of data from five animals. The enzyme activities are expressed as: SOD—units per milligram protein; CAT—micromoles of H_2_O_2_ consumed/min/mg protein; GPx—micrograms of reduced glutathione consumed/min/mg protein. ^a^Difference with the control is statistically significant (p < 0.001); ^b^Difference between treatment groups is statistically significant (p < 0.05)

**Fig. 8:**
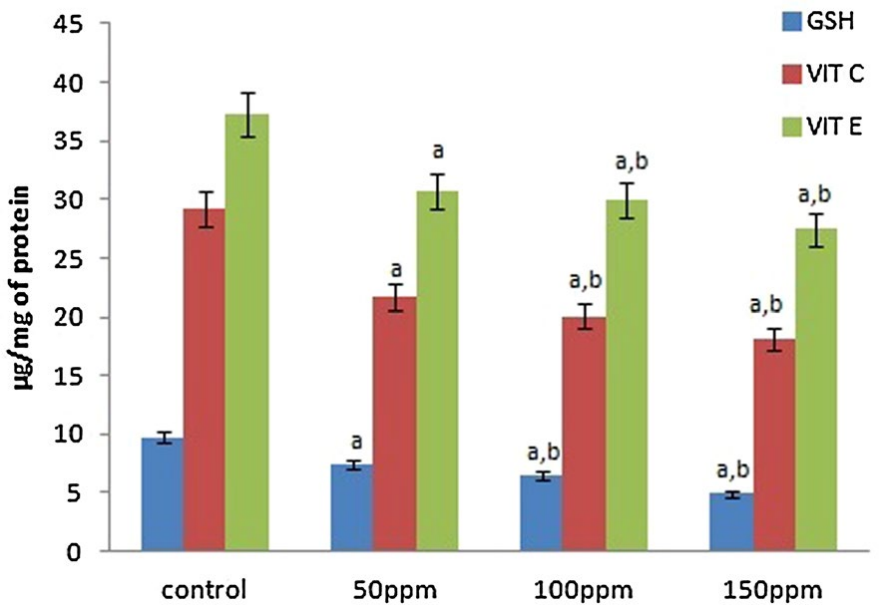
Levels of non-enzymatic antioxidants in testis tissue of Syrian hamster. Each value represents the mean ± SD of data (μg/mg of tissue) from five animals. ^a^Difference with the control is statistically significant (p < 0.001); ^b^Difference between treatment groups is statistically significant (p < 0.05)

**Fig. 9:**
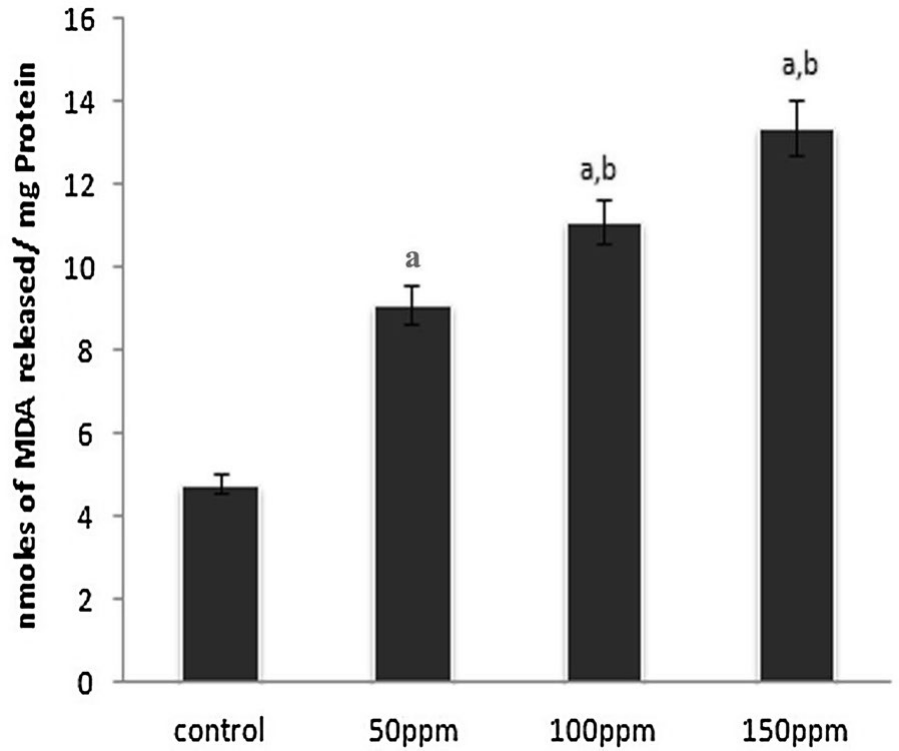
Level of malondialdehyde (MDA) in testis tissue of Syrian hamster. Each value represents the mean ± SD of data (nmoles of MDA formed/mg protein) from five animals. ^a^Difference with the control is statistically significant (p < 0.001); ^b^Difference between treatment groups is statistically significant (p < 0.05)

**Fig. 10:**
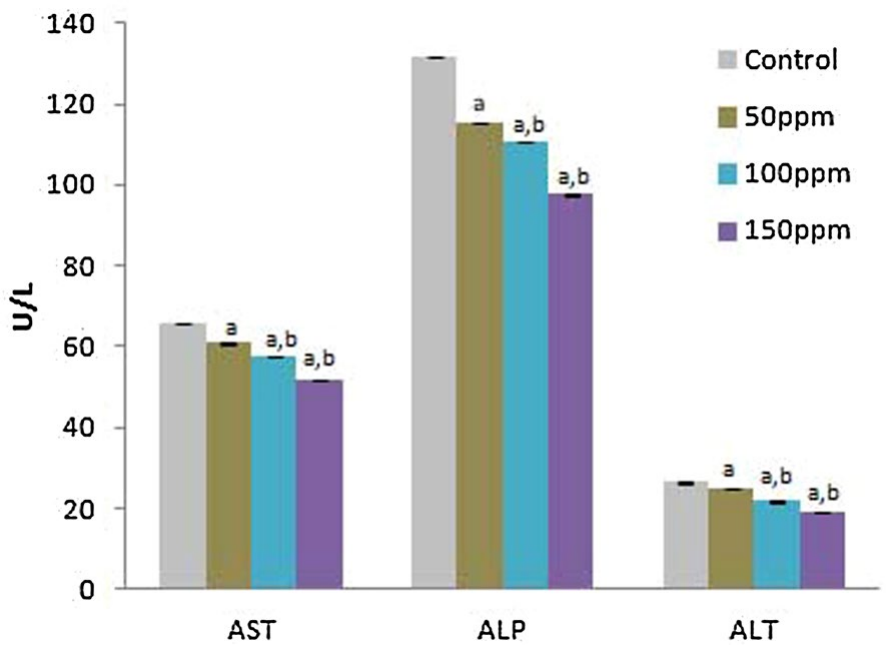
Activity levels of serum marker enzymes of tissue injury (AST, ALT and ALP) in Syrian hamster. Each value represents the mean ± SD of data (U/l) from five animals. ^a^Difference with the control is statistically significant (p < 0.001); ^b^Difference between treatment groups is statistically significant (p < 0.05)

The mean activities of serum ALT, AST and ALP were found to be significantly (p < 0.001) decreased in Group II (50 ppm), Group III (100 ppm) and Group IV (150 ppm) hamsters compared to Group I (control) hamsters, in a dose-depended manner. Among the different treatment groups the values were significantly (p < 0.05) different (Figs.  4, 5, 6, 7, 8, 9, 10).

### Effects of TBT on serum biochemical parameters

**Fig. 11:**
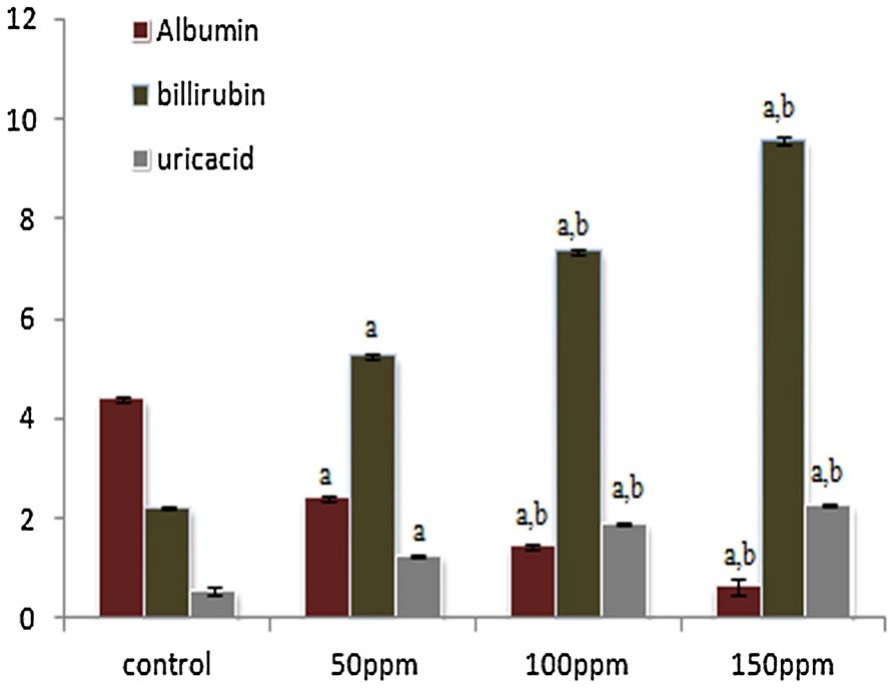
Effect of TBT on serum albumin, bilirubin and uric acid in Syrian hamster. Each value represents the mean ± SD of data (mg/dl) from five animals. ^a^Difference with the control is statistically significant (p < 0.001); ^b^Difference between treatment groups is statistically significant (p < 0.05)

**Fig. 12:**
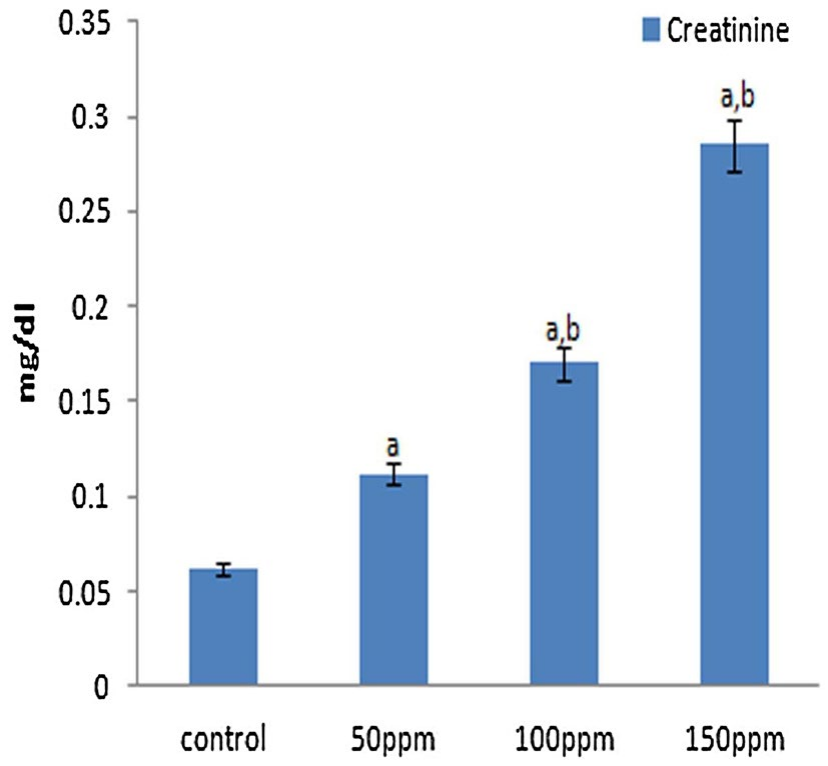
Effect of TBT on serum creatinine in Syrian hamster Each value represents the mean ± SD of data (mg/dl) from five animals. ^a^Difference with the control is statistically significant (p < 0.001); ^b^Difference between treatment groups is statistically significant (p < 0.05)

**Fig. 13:**
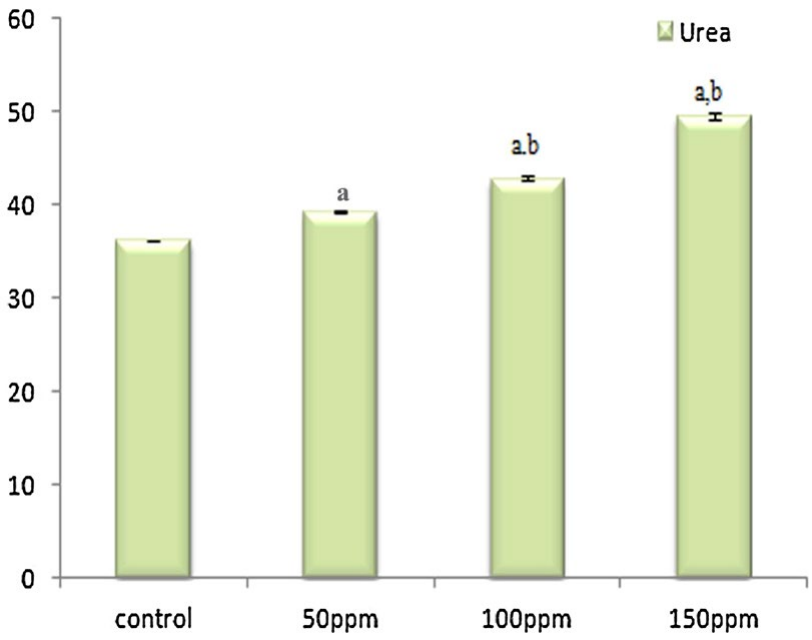
Effect of TBT on serum urea in Syrian hamster. Each value represents the mean ± SD of data (mg/dl) from five animals. ^a^Difference with the control is statistically significant (p < 0.001); ^b^Difference between treatment groups is statistically significant (p < 0.05)

The concentration of serum albumin was found to be significantly (p < 0.001) decreased in all three TBT-treated group of hamsters compared to control. On the other hand the mean levels of bilirubin, uric acid, creatinine and urea increased to significant levels (p < 0.001) compared to control hamsters. The values between treatment groups in each parametric case was also significantly (p < 0.05) different (Figs. 11, 12, 13)

### Histopathological changes in the testis, kidney and liver

**Fig. 14:**
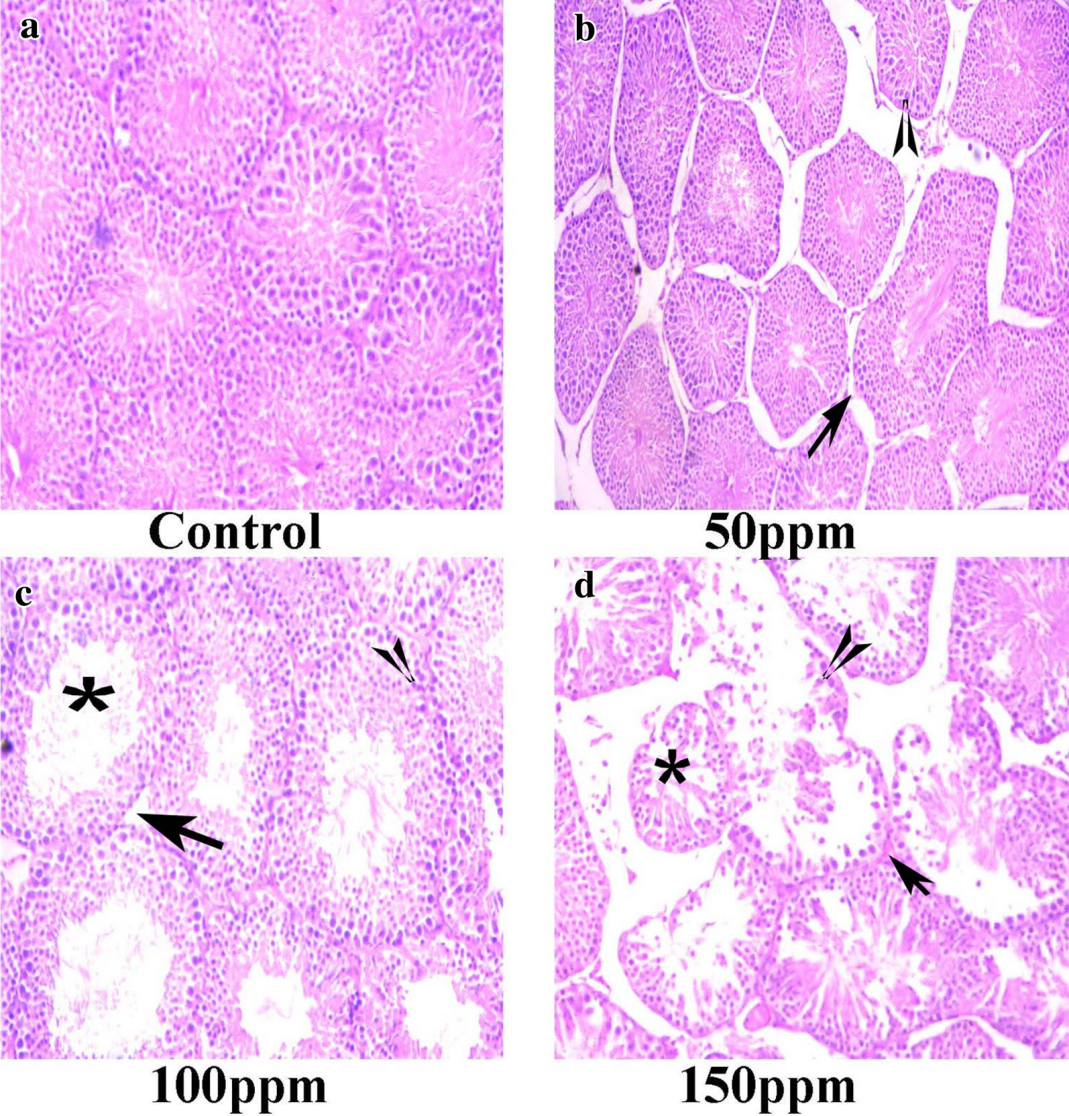
Section of testis tissue of Syrian hamster. Hematoxylin-eosin staining; ×200. **a** Control testis showing normal organization of seminiferous tubules, with normal spermatogonia, spermatocytes, spermatids, spermatozoa and Sertoli cells. **b**–**d** TBT treatment groups at 50, 100 and 150 ppm concentrations showing abnormalities such as disruption of spermatogonia (*arrow head*), regression of seminiferous tubules (*asterisk*), degeneration of seminiferous epithelium, and edematous Leydig cells (*arrow*)

**Fig. 15:**
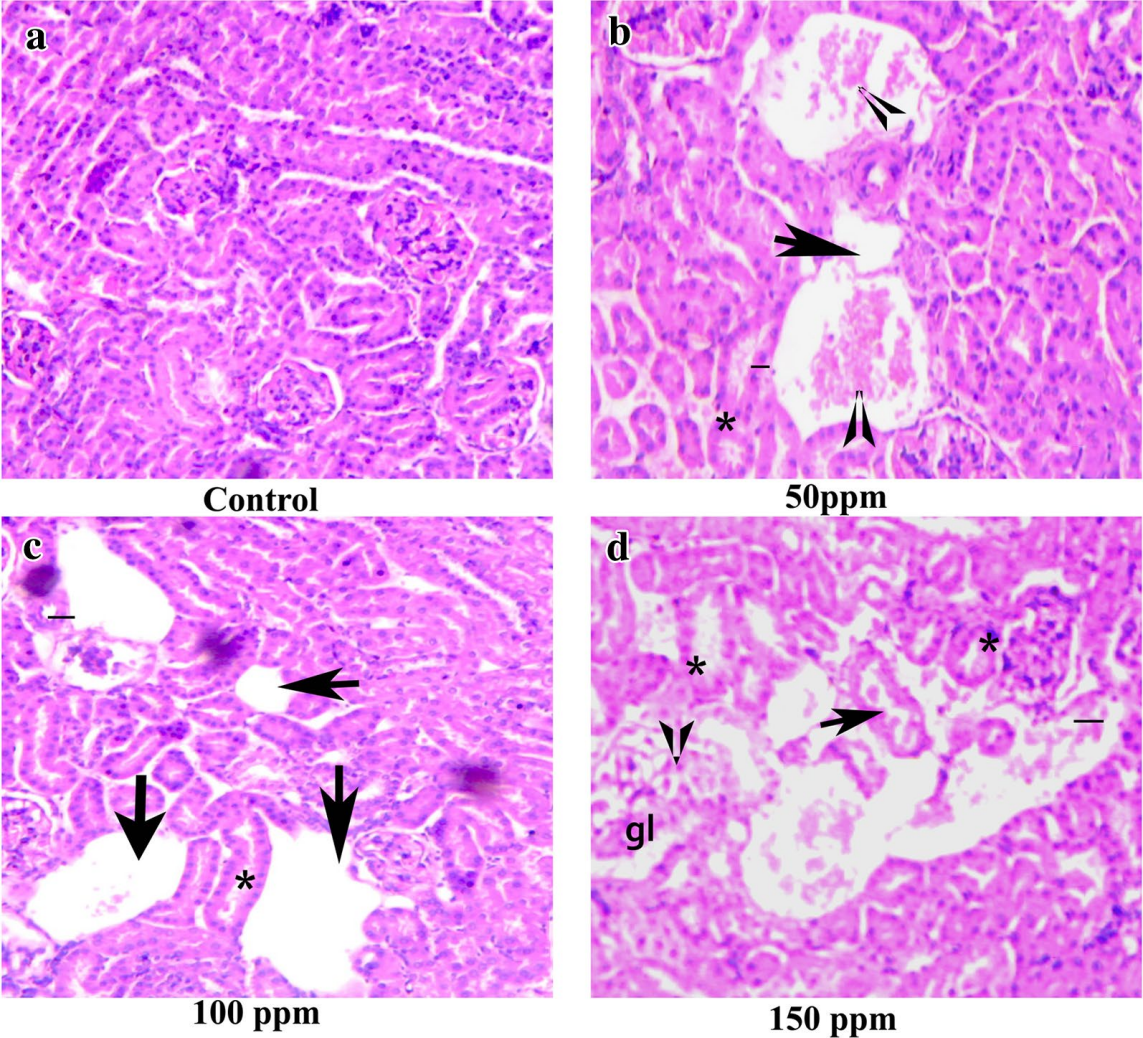
Section of kidney tissue of Syrian hamster. Hematoxylin-eosin staining; ×200. **a** Control kidney showing normal renal tubules and glomerulus. **b**–**d** TBT-treated at 50, 100 and 150 ppm, showing congested renal vein (*arrow*), hemorrhagic foci with tubular necrosis (*single line*), dilatation, cellular debris accumulation in the tubular lumen (*arrow head*), scattered hemorrhages (*asterisk*), intertubular fibrosis and swelling in the lining endothelium of the glomerulus tuft (gl)

Control testis showed normal testicular histo-architecture. The seminiferous tubules possessed normal spermatogonia, spermatocytes, spermatids, spermatozoa and Sertoli cells. TBT treatment resulted in abnormalities such as disruption of spermatogenesis, regression of seminiferous tubule diameter, necrosis of Leydig cells, and degeneration of seminiferous epithelium (Fig. 14). Section in the kidney of hamsters treated with TBT at various doses of 50,100 and 150 ppm showed congested renal vein, hemorrhagic foci with tubular necrosis, cellular debris accumulation in the tubular lumen, scattered hemorrhages, inter-tubular fibrosis and swelling in the lining endothelium of the glomerulus tuft whereas section of the kidney of a control animal showed normal renal tubules and glomeruli (Fig. 15).

**Fig. 16:**
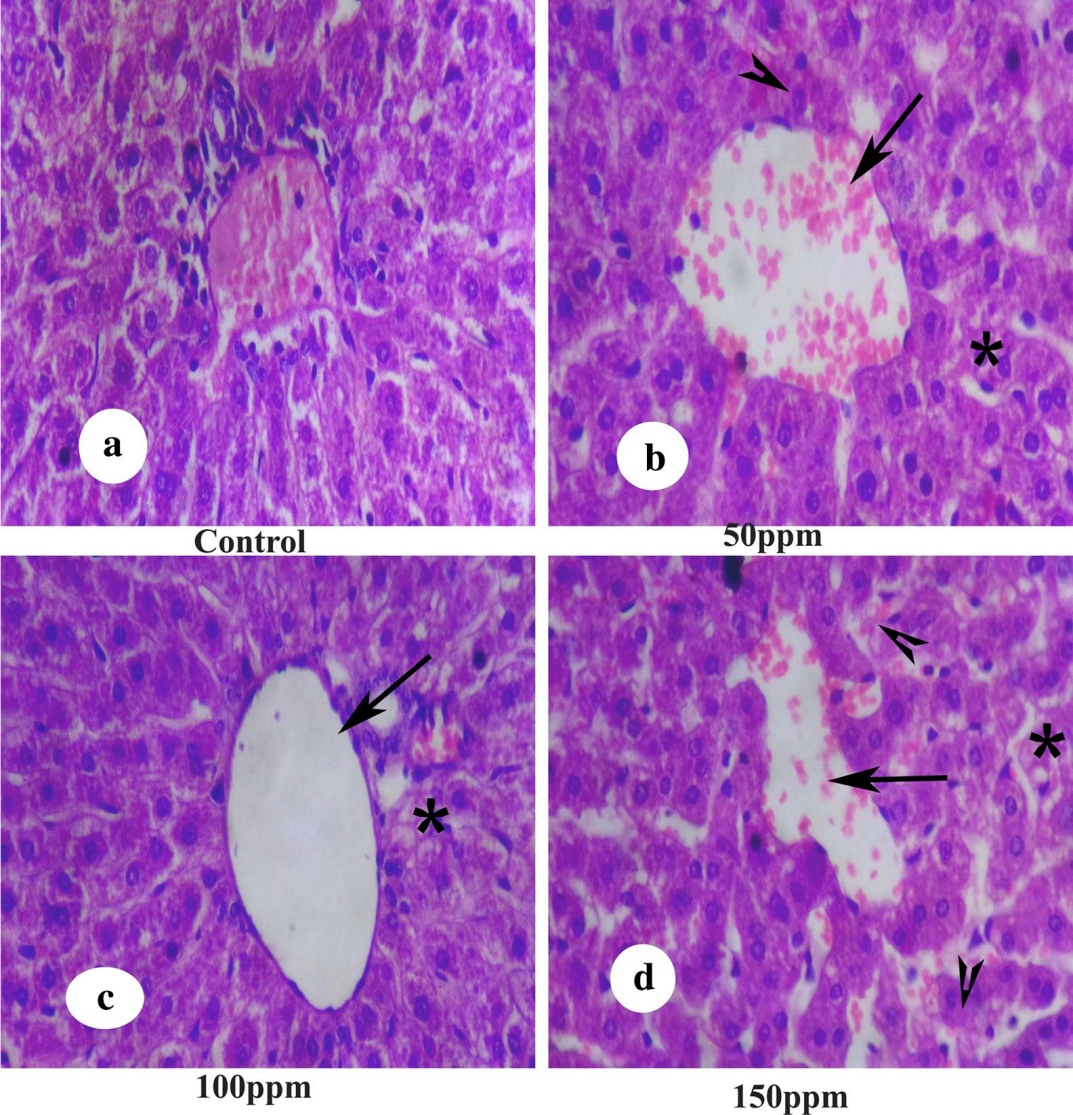
Section of liver tissue of Syrian hamster. Hematoxylin-eosin staining; ×200. **a** Control liver, showing normal cellular cords and hepatocytes; **b**–**d** TBT treated at 50, 100 and 150 ppm, showing severe hepatocyte damage as manifested by noticeable fat vacillation or empty spaces (*arrow head*), dilated hepatic veins (*arrow*), and necrosis of hepatocytes (*asterisk*)

The liver histology showed normal hepatocytes in the control group whereas the TBT treated group showed severe hepatocyte damage which was manifested by noticeable fat vacillation or empty spaces; the hepatic veins were clearly dilated and the hepatocyte cells were disintegrated or necrotic (Fig. 16).

## Discussion

TBT, a known endocrine disruptor, is of major concern in the current context of declining male fertility (Kanimozhi et al. 2014). The problematic aspect of TBT is that it accumulates in sediments and may cause long-term adverse effect in aquatic organisms and through the food chain to humans (Aline et al. 2014). Once inside the cell TBT causes increased production of ROS, this leads to lipid peroxidation and cell death (Ishihara et al. 2012). The enzymatic and non-enzymatic antioxidant defense systems that include SOD, CAT, GPX, vitamin C, vitamin E and GSH become deficient on TBT-exposure. The consequent uncontrolled generation of ROS during TBT-induced toxicity culminates in tissue damage, leading to impaired cellular function, alterations in the physiochemical properties of cellular membranes and reduced enzyme activities (Li et al. 2016; Huang et al. 2005a, b).

In the present study, the activities of enzymatic antioxidants SOD, CAT, and GPx were significantly decreased in TBT-treated male Syrian hamster. SOD is responsible for the dismutation of superoxide radical to H_2_O_2_, which is deactivated by the mutual action of CAT and GPx (Murugesan et al. 2005). The reduction in enzyme synthesis or inactivation of the enzyme during TBT exposure may reduce the activities of SOD and CAT in liver, kidney and testis. Alteration in these enzyme activities may possibly push the cell into oxidative stress. Oxidative stress has been identified as one of the major factors that affect male fertility status, and various environmental toxicants are reported to induce production of ROS, thereby causing oxidative changes in tissues (Senthil Kumar et al. 2004). Ishihara et al. (2012) have suggested that TBT stimulates excessive oxygen free radical generation leading to lipid peroxidation, oxidative stress and damage of cellular macromolecules like proteins, lipids and nucleic acids.

The levels of non-enzymatic antioxidants GSH, Vit C and Vit E were significantly decreased in tributyltin-treated male Syrian hamster. Vitamin C (Ascorbate) is a potent water soluble scavenger of ROS and nitrogen species including hydroxyl radical, peroxyl radical, superoxide anion, nitrogen dioxide as well as non-radical species such as hypochlorous acid, ozone and singlet oxygen. It has been reported that even a marginal vitamin C deficiency would result in intracellular oxidative damage (Rekha et al. 2011).

The antioxidative function of vitamin E is in view of its action at the membrane phospholipid bilayer to break the chain reaction initiated by the hydroxyl radical. Thus, vitamin E protects the cells against lipid peroxidation, where free radical assault on fatty acids causes structural damage to membranes and results in the formation of cytotoxic secondary products such as MDA (Burton and Traber 1990). An increase in MDA concentration was observed in TBT-treated hamsters compared to control, which is associated with tissue damage, an inference supported by histopathological analysis of liver, kidney and testis. The elevation in urea and creatinine levels in hamsters exposed to TBT is a significant marker of renal dysfunction. The reflection in serum urea may be due to metabolic disturbances like renal function and cationic balance. According to Koh et al. (2012) the increase of urea concentrations in serum may be due to toxic effect towards liver function, as urea is the end product of protein catabolism. Increase in total bilirubin was observed in TBT treated animals which may be due to reduced liver uptake, conjugation or increased bilirubin production from hemolysis (Muraca et al. 1987). Some of the essential functions of the liver include detoxification of bilirubin, epimerization of galactose to glucose as uridine-5-phosphate derivatives and synthesis of protein (albumin) and prothrombin. Many of these functions are disrupted when a hepatotoxic substance damages liver cells due to lipid peroxidation and other oxidative processes (Bigoniya et al. 2009).

Enzymes such as ALT, AST and ALP serve as markers of oxidative stress. The hepatic damage caused due to TBT exposure is evidenced through the released cytoplasmic enzymes such as AST and ALT into circulation. Reduced activity of AST and ALT directly manifested by noticeable fat vacillation or empty space, the hepatic veins were clearly dilated and the hepatocytic cells were disintegrated, and necrosed. Massive local hemorrhage of the renal tissues, necrosis and/or atrophic glomeruli, kidney tubules, glomerular capsule and tubules dilatation were observed in histology of kidney. Serum bilirubin was significantly increased in 50, 100 and 150 ppm treated hamsters compared to control which might indicate regurgitation of bile from obstruction within the liver resulting from damage or inflammation caused by TBT.

This study shows that 65 day oral exposure of TBT altered the liver, kidney and testicular functions in hamster. Also, significantly decreased level of ALP was noted in TBT exposed hamster compared to normal hamster. ALP is the testicular tissue function marker; hence, decrease in ALP level directly associated with histological examination of the testis that revealed atrophy of seminiferous tubules, loss of spermatogenic cell layers, absence of the spermatozoa in the lumen and degeneration of Leydig cell in TBT-treated hamster, whereas the control group showed active spermatogenesis with all the germ cells such as spermatogonia, primary and secondary spermatocytes, spermatids and sperms in the lumen. The increased level of bilirubin in some cases is due to renal failure (Mizote et al. 2008). Renal damage observed in hamster exposed to tributyltin reflects upon renal pathology. There was a significant decrease in serum albumin which might reflect liver necrosis. Apart from this, intervention with albumin-synthesizing process in the liver might have resulted in inflammation that reflected in decrease of albumin levels. We found a significant decrease in serum levels of urea and creatinine due to toxic effects of TBT correlated with renal dysfuction. The toxicity induced by TBT was marked by antioxidant and histological alterations in hamster, which relate with change in the levels of creatinine, bilirubin, urea and albumin.

In conclusion, TBT exposure results in free radical-mediated toxicity in the liver, kidney and testis of Syrian hamster. The higher fabrication of the free radicals or decreased function of the defense system may play a role in the renal, hepatic and testicular toxicity of TBT.
